# Magnetic hard gap due to bound magnetic polarons in the localized regime

**DOI:** 10.1038/srep42224

**Published:** 2017-02-08

**Authors:** Gaurab Rimal, Jinke Tang

**Affiliations:** 1 Department of Physics & Astronomy, University of Wyoming, Laramie, Wyoming 82071, USA

## Abstract

We investigate the low temperature electron transport properties of manganese doped lead sulfide films. The system shows variable range hopping at low temperatures that crosses over into an activation regime at even lower temperatures. This crossover is destroyed by an applied magnetic field which suggests a magnetic origin of the hard gap, associated with bound magnetic polarons. Even though the gap forms around the superconducting transition temperature of lead, we do not find evidence of this being due to insulator-superconductor transition. Comparison with undoped PbS films, which do not show the activated transport behavior, suggests that bound magnetic polarons create the hard gap in the system that can be closed by magnetic fields.

Metal-insulator transitions (MIT) due to disorder or electron-electron interaction have been at the center of condensed matter physics research. Without the interaction, the scaling theories of localization have been well established to provide the physical picture of disordered systems in various dimensions^1^,^2^. When the interaction is important and the interplay between the two must be considered, the physics becomes more intriguing. On the insulating side of MIT, disorder-induced localized states reduce the charge screening, and the Coulomb interaction between electrons cannot be ignored. Efros and Shklovskii have shown that the Coulomb interaction in the insulating state opens a parabolic soft gap in the density of states (DOS)^3^ and the transport follows Efros and Shklovskii (ES) variable range hopping (VRH).

An interesting and yet unresolved issue is the opening of a hard gap (HG) as demonstrated in the crossover from the VRH to activated transport behavior when the temperature is lowered in some systems. Due to electron-electron correlations, the DOS can be depleted below a certain temperature and a HG is formed close to the Fermi level^4^. Several possible mechanisms may be responsible to the HG formation. Electronic (lattice) polarons can result in the HG at low temperature^5^,^6^. HGs may also be of magnetic origins associated with the exchange coupling as found in Mn doped CdTe and B doped Si^7^,^8^. This type of HG is characterized by its closure with an applied magnetic field. On the other hand, there have been reports of HGs in magnetically doped semiconductors that are not influenced by the magnetic field^9^. In addition, HGs may arise on the insulating side of the superconductor-insulator transition^10^,^11^,^12^. Depending on the mechanisms of the HG formation, low temperature measurements may or may not reveal the crossover from ES VRH hopping to transport of activation type^7^,^13^,^14^. A comprehensive theory for HG formation and its correlation to the transport behaviors is currently lacking.

In strongly localized diluted magnetic semiconductors (DMS), the spins from the localized states may exchange couple to and align the spins of the transition metal dopants that can lead to the formation of a bound magnetic polaron (BMP)^15^,^16^,^17^,^18^. BMP systems often exhibit long range magnetic ordering^19^, which is determined by the percolation of the magnetic polarons in the systems^20^,^21^ and may lead to spintronic applications. In the dilute limit, BMPs hop from one site to another. The transport of BMP typically follows VRH, sometimes with modification^22^. It was argued^7^ that adding a bare electron to the system costs the energy of spin relaxation due to the formation of BMP at the final site of the hop. Removing it from the initial site also costs energy due to the decrease in the binding energy after the originally aligned dopant spins thermalize. As a result, a HG forms in the electronic DOS and an activated character is developed at low temperatures. This seems to be the case for Mn doped CdTe, B doped Si, and Mn doped Si^7^,^8^,^23^. We should note that theory establishing the correlation between spin-spin interaction and HG has yet to be developed^22^,^24^. Some suggest that the presence of a spin glass (or other types of disordered) background state is necessary for the activated behavior^14^,^23^,^25^, and others show inconsistency between the calculated and experimental gaps that can only be resolved if the hopping time is assumed to be shorter than the BMP formation time^26^. In another word, there needs to be sufficient time for the BMP to form completely.

In this paper, we study the low temperature magnetotransport properties of Mn doped PbS (MnPbS), which shows the crossover from ES VRH to activated transport behavior at low temperature. We confirm the magnetic nature of the HG - an applied magnetic field closes the HG. Although there have been many reports of III–V and II-VI based DMS^27^, IV–VI DMS are rarely seen in literature. It is also noteworthy that PbS based DMS, mainly nanocrystals, have been studied for their magneto-optic properties^28^,^29^,^30^,^31^,^32^. It is found that sp-d coupling between the carriers and Mn dopants leads to a giant Zeeman splitting, and a large effective *g*-factor and sign change in the circular polarization are observed upon Mn doping^28^. A sign reversal in the exciton g-factor with increasing Mn concentration has also been inferred based on low Mn concentration data^30^.

## Results

**Figure 1:**
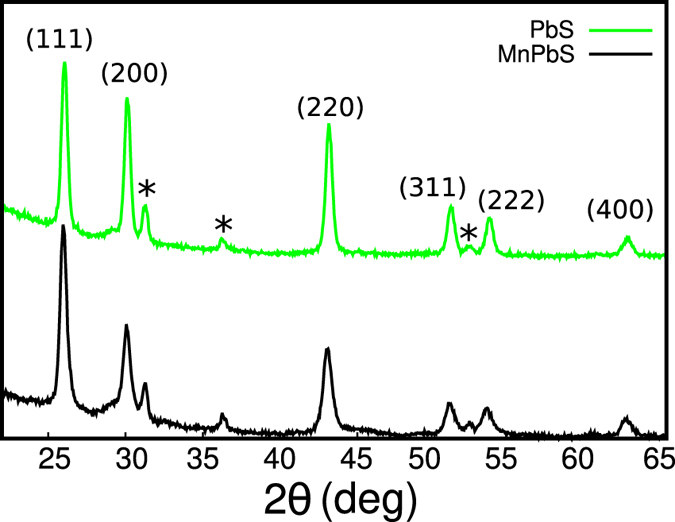
XRD for the doped and undoped films. Pb clusters have also been identified in the film matrix (marked with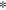).

PbS and MnPbS films were deposited using pulsed laser deposition onto a quartz substrate. X-ray diffraction (XRD) patterns showed that the samples crystallize in the fcc rocksalt structure (Fig. 1). Pb impurity peaks were also seen in XRD which should exist as metallic clusters. For the doped films, it was seen that the Mn concentration would always be around 4 at.%, pointing to a solubility limit of the dopant in the PbS matrix. Thus our doped films were in Pb_0.96_Mn_0.04_S configuration.

**Figure 2:**
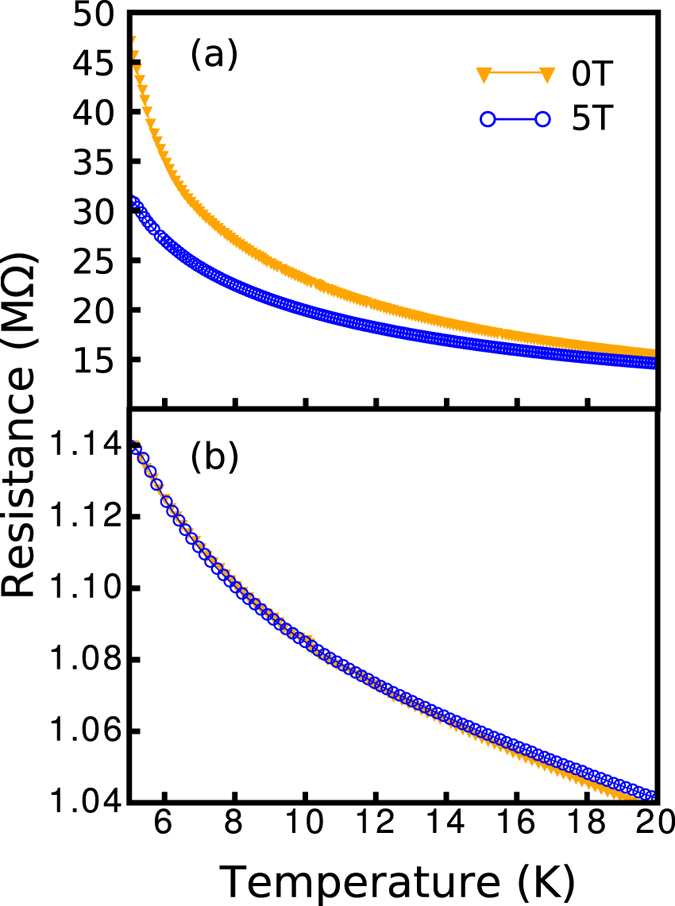
Temperature dependence of resistance at B = 0 T, 5 T for the (**a**) doped (**b**) undoped films.

**Figure 3:**
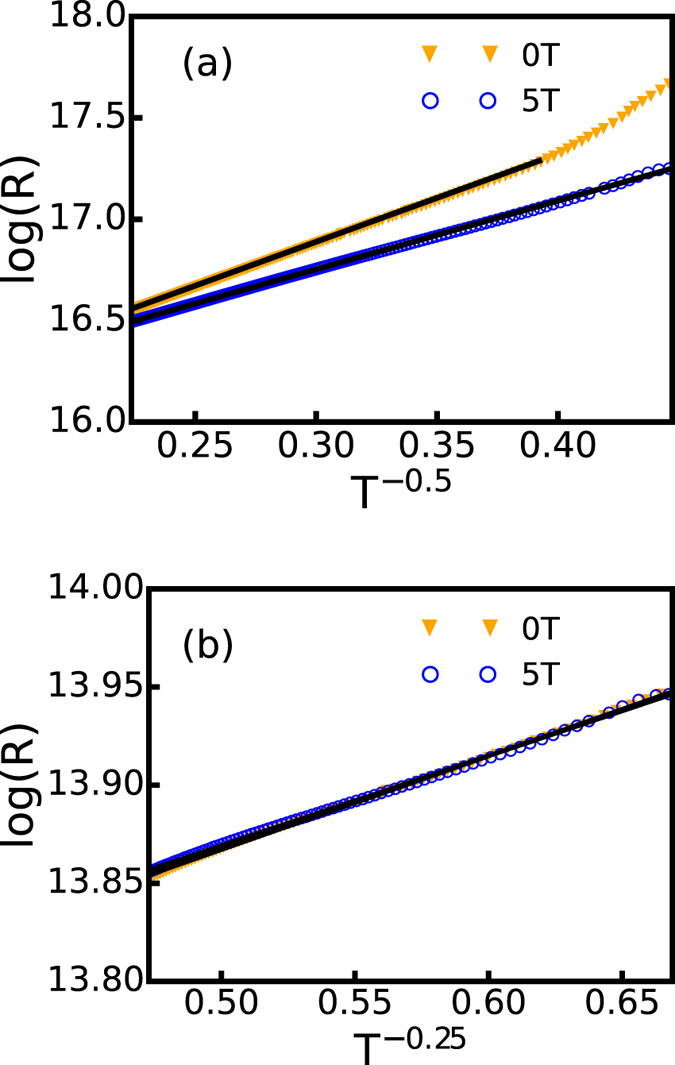
The logarithm of resistance plotted against *T*^−*β*^ to show the effect of field on the low temperature transport mechanism of localized carriers. The straight lines are fit using equation 1. (**a**) MnPbS, 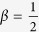 (**b**) PbS, 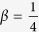.

For transport meaurements, the I–V responses were ohmic for all temperatures, and both AC and DC measurements showed similar results. Figure 2 shows the temperature dependence of resistance of the doped and undoped PbS. At lower temperatures, the resistance rises with decreasing temperature faster than at moderate temperatures for the doped films. On further analysis (see Fig. 3), we observe that at lower temperatures, its transport mechanism changes from ES VRH to activated behavior. Specifically, the temperature dependence of the resistance of the samples can be described by


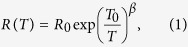


where the exponent *β* indicates the types of transport. For the undoped films, VRH of Mott type 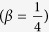 continues down to the lowest measured temperatures, showing transport that differs from the doped. For the doped films, it is found that the ES VRH 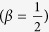 type transport crosses over to the activation type (*β* = 1) below 8 K. In other words, the soft gap hardens as the temperature is lowered. An applied field of 5 T destroys the HG in the doped sample and reverts the system back to the ES VRH regime. This suggests that the hard gap is magnetic in nature. As will be discussed later, the HG is associated with BMP, which can be closed by the magnetic field. As a matter of fact, above the crossover temperature, the 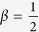 behavior is followed both with and without a magnetic field except that T_0_ is field dependent, and we find that 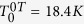 and 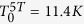.

**Figure 4:**
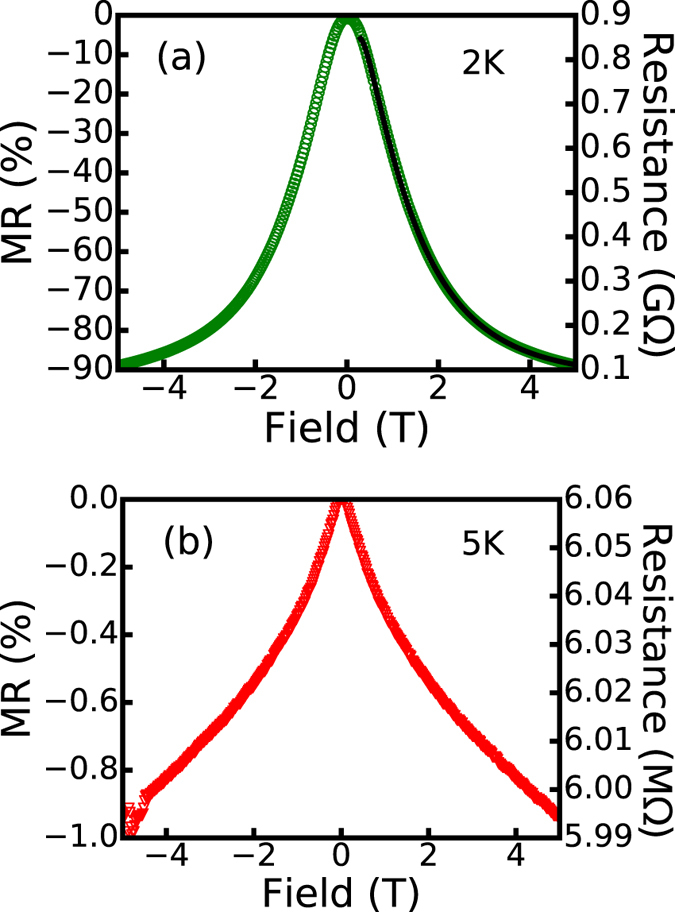
(**a**) MR behavior of MnPbS at 2 K. Solid black line is the fit at 2 K using KS model (eq. 2). (**b**) MR of PbS at 5 K.

**Figure 5:**
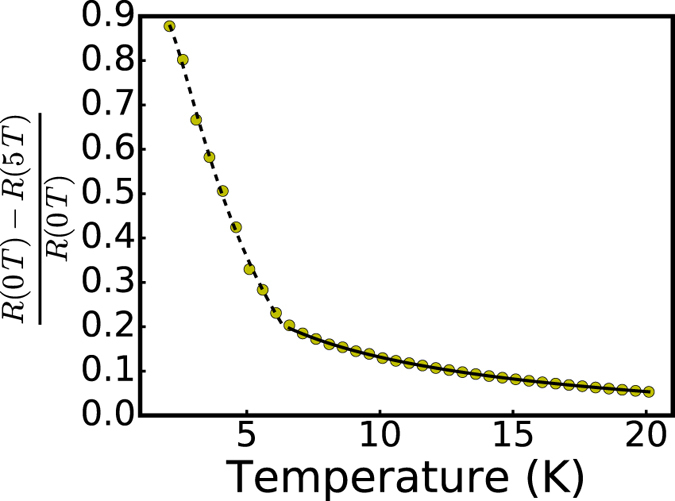
The MR in the two temperature regions, divided by the crossover temperature, is described with different functions (shown as dashed and solid lines). See text for details.

Consistent with the field dependence of the HG, a giant negative magnetoresistance (MR) at B = 5 T (≈90% at 2 K and ≈40% at 5 K) is found in the material, which is different both in magnitude and field dependence from its undoped counterpart. Though the undoped films also show negative MR, its magnitude is about 1% in the same field at T = 5 K. The field dependence of the MR is also very different between the two samples (see Fig. 4). The behavior of the doped films is consistent with the magnetic polaron model developed by Kaminski and Das Sarma (KS)^20^, which has the form


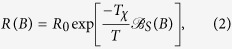


where T_*χ*_ is a characteristic temperature that is related to the exchange constant, and 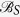 is the Brillouin function. From the fit, we find that *T*_*χ*_ = 5.5 K. It should be noted that we have also kept the spin S within the Brillouin function as a fitting parameter and found that 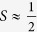. The value of the HG is determined independently from the fit to the R-T curve below the crossover temperature and is found to be Δ = 6.9 K. It should also be noted from Fig. 5 that the the MR behavior changes around the crossover temperature, which is due to the shift from 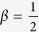 to *β* = 1.

## Discussion

The resistivity of Mn doped films is much higher than the undoped films. A possible reason for the increase can be attributed to the formation of BMP. Doped films show ES type hopping while undoped films show Mott hopping (see Fig. 3). In VRH, the Coulomb interaction between electrons generally leads to a change from Mott to ES type hopping with the opening of a soft gap. In the case of BMP, a similar situation may occur due to spin-spin interaction^8^,^22^. It is most likely that the resistivity of the system increases due to the increased difficulty of hopping as the carriers need to align the spins of the Mn dopants during such a process. At the same time, the transport switches to ES type from Mott hopping. A number of DMS systems have been reported to exhibit ES type hopping as a result of spin-spin interaction and BMP formation^8^,^33^.

The temperature dependence of the resistance shows a crossover from hopping to activation mechanism at low temperatures, implying that the DOS vanishes for MnPbS. Yet, an external magnetic field reverts this behavior, suggesting spin-spin correlation rather than charge-charge correlation between electrons is responsible for the HG formation^5^,^7^. The 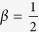 VRH behavior above the crossover temperature suggests the hopping transport of BMP in the doped samples. The presence of BMP is supported by the negative MR of moderate values for T > 10 K (see Fig. 5). Intuitively, it is not hard to understand that hopping of BMP become difficult at low temperatures due to the cost of energy to form and relax the BMP as they hop, which results in the HG below the crossover temperature. The question that we do not have a definitive answer to at this moment is what is the origin of the energy of spin relaxation and whether the HG is associated with any spin glass or disordered states that forms in the background that hinders the hopping of the BMP. AC magnetic susceptibility measurements of the doped samples do not reveal any anomaly near the crossover temperature. Nevertheless, when a high magnetic field is applied, these frozen spins in the spin glass or disordered states are relaxed^34^, thereby assisting the carrier hopping and BMP formation. This leads to the closure of the HG and the system returns to the VRH regime.

The crossover temperature happens to be close to the superconducting transition temperature of lead. HGs can also be observed on the insulating side of superconductor-insulator transition (SIT) in granular and disordered superconducting films^10^,^11^,^12^. These systems exhibit a fast increase of the resistance below the superconducting transition temperature and, in highly insulating samples, a crossover into the activation regime from VRH regime. We can rule out the possibility of superconducting Pb clusters being the origin of the HG in our samples because we do not observe such effect in the undoped films even though similar amount of Pb clusters are present in both films. It is the Mn dopants, not the superconducting lead, that result in the observed behavior. There are additional evidence that rules out the superconducting gap scenario. In SIT systems close to the critical temperature, MR is generally positive at low fields which changes to negative at higher fields as a result of the superconducting gap being suppressed by the field^11^,^35^,^36^. However, the MR associated with magnetic HGs is always negative as seen in the case of Mn doped PbS. In addition, the negative MR due to the suppression of the superconducting gap typically shows a parabolic B^2^ dependence that persists up to a relatively high field of several tesla^37^ which is clearly not the case here (see Fig. 4(a)). More importantly, the MR of our samples fits well with the KS model. The characteristic temperature T_*χ*_ obtained from the field dependence fit (T_*χ*_ = 5.5 K), which roughly measures the exchange coupling strength of the BMP, is reasonably close to the HG obtained from the temperature dependence of the resistivity (Δ = 6.9 K), demonstrating the intimate connection between the HG and BMP.

As mentioned, an interesting aspect of the HG regime is the observation of giant negative MR. From Fig. 5, one can see that the negative MR increases rapidly below the crossover temperature, which roughly scales with the temperature dependence of the resistivity. Attempt to fit the temperature dependence of the MR resulted in a mixture of VRH 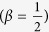 and activated behavior (dashed line in Fig. 5), which is expected from the temperature dependence of the resistivity. The temperature dependence of MR below the crossover temperature also behaves differently compared to the MR above it. Above the crossover temperature, the MR can be approximated by the hopping model (eq. 1, with 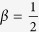) since hopping is ES type regardless of the applied field (solid line in Fig. 5). The MR versus field curve below the crossover fits well to the KS BMP model. The fit produces a characteristic temperature T_*χ*_ that is consistent with the hard gap, confirming the role of the BMP in the HG formation. The undoped films also have negative MR but the magnitude is much smaller. This negative MR is likely related to the quantum interference effect^38^,^39^. In addition, the R versus T dependence is of Mott type, not ES type, which continues through the lowest temperature measured.

In summary, the low temperature transport properties of MnPbS has been investigated which shows the crossover from a soft gap to a HG in the DOS. Localized electron at a given site polarizes the Mn dopants around it via exchange coupling and forms a BMP. The hopping of BMP is hindered at low temperature which opens the HG. An applied magnetic field reverts the hard gap to the soft gap confirming the BMP origin of the HG. This is manifested as the change in transport from VRH to activation regime below the crossover temperature along with the giant negative MR. Question remains open as to the origin of the energy of spin relaxation that leads to the HG. It is possible that a spin glass or disordered state forms in the background and the associated slow spin relaxation hinders the hopping of the BMP; however this escaped the detection in our experiments. Doped PbS is also unique in that strong spin-orbit coupling may open a gap^40^. Further studies are needed to answer such questions.

## Methods

Lead acetate (99.999%, Sigma Aldrich), sodium sulfide (99.99%, Sigma Aldrich) and manganese(II) chloride (99.999%, Alfa Aesar) were combined in right proportions in aqueous solution and the precipitate thoroughly cleaned and dried. X-ray diffraction showed the resulting powder to be in PbS rocksalt phase, and no trace of MnS was found. The powder was then ground and cold pressed to make a target for the PLD, which was mounted into the PLD chamber and used thereafter. The Mn concentration in the target was ~10 at.% as obtained from energy dispersive x-ray spectroscopy (EDX). The same method was used to prepare PbS target but without the MnCl_2_ source.

For PLD, a Lotis-TII Nd:YAG laser operating at fourth harmonic of 266 nm was used. Quartz and Si substrates were mounted into the chamber which was pumped down to a pressure better than 2 *μ* Torr. The substrates were then heated to 350 °C. The laser with a pulse energy of 280 mJ and repetition rate of 10 Hz was focused onto the target which was kept at a distance of about 5 cm from the substrate. The deposition was carried out for 60 mins, and the films further annealed for 60 mins.

The films were characterized using XRD (Rigaku smartlab), scanning electron microscopy and EDX (FEI quanta FEG 450). After characterization, square shaped devices in four-probe configuration were mounted inside a physical property measurement system (PPMS). Transport measurements were then performed using SR830 and SR850 lockin amplifiers for low frequency AC and Agilent 4156C for DC measurements.

## Additional Information

**How to cite this article**: Rimal, G. and Tang, J. Magnetic hard gap due to bound magnetic polarons in the localized regime. *Sci. Rep.* **7**, 42224; doi: 10.1038/srep42224 (2017).

**Publisher's note:** Springer Nature remains neutral with regard to jurisdictional claims in published maps and institutional affiliations.
